# Phototherapy-Associated Changes in Ionized Calcium Among Neonates Born at ≥35 Weeks of Gestation

**DOI:** 10.7759/cureus.116570

**Published:** 2026-09-20

**Authors:** Sahil Chauhan, Vijay K Singh, Priyanka Singh, Rachana Bhatnagar

**Affiliations:** 1 Pediatrics and Neonatology, Baba Raghav Das Medical College, Gorakhpur, IND

**Keywords:** hypocalcemia, ionized calcium, jaundice, neonatal hyperbilirubinemia, neonates, phototherapy

## Abstract

Background

Phototherapy is the standard treatment for clinically significant unconjugated neonatal hyperbilirubinemia. Although generally safe and effective, phototherapy has been associated with alterations in calcium homeostasis. The magnitude and clinical relevance of phototherapy-associated reductions in ionized calcium (iCa) remain uncertain, particularly among neonates born at ≥35 weeks of gestation.

Objective

To evaluate changes in iCa before and after phototherapy in neonates born at ≥35 weeks with unconjugated hyperbilirubinemia.

Methods

This prospective cohort study included 145 neonates born at ≥35 weeks who received phototherapy for at least 24 hours for unconjugated hyperbilirubinemia at the Department of Pediatrics, Baba Raghav Das (BRD) Medical College, Gorakhpur, India, from August 2021 to August 2022. Ionized calcium was measured immediately before the initiation of phototherapy and after 24 hours of phototherapy using an ion-selective electrode (ISE) method and was categorized as <1.20, 1.20-1.40, and >1.40 mmol/L. Changes in the paired distribution of iCa categories were assessed using the Stuart-Maxwell test of marginal homogeneity.

Results

The mean gestational age was 36.93 ± 1.49 weeks; 64 (44.1%) neonates were born before 37 weeks, and 82 (56.6%) were male. Before phototherapy, 12/145 (8.3%) neonates had iCa <1.20 mmol/L, compared with 54/145 (37.2%) after 24 hours of phototherapy, representing an absolute increase of 28.9 percentage points. Among 133 neonates with baseline iCa ≥1.20 mmol/L, 44 (33.1%) developed iCa <1.20 mmol/L after 24 hours of phototherapy. The proportion with iCa >1.40 mmol/L decreased from 109/145 (75.2%) before treatment to 30/145 (20.7%) after 24 hours. The overall shift in paired iCa categories was statistically significant (Stuart-Maxwell χ² = 83.808, df = 2, p < 0.001).

Conclusion

Phototherapy was associated with a significant downward shift in iCa among neonates born at ≥35 weeks of gestation. Among neonates without low iCa at baseline, 33.1% developed concentrations <1.20 mmol/L after 24 hours of phototherapy. These findings demonstrate an important biochemical association; however, further studies are needed to determine its clinical significance and identify neonates who may benefit from targeted iCa monitoring.

## Introduction

Neonatal hyperbilirubinemia is among the most common clinical conditions encountered during the first week of life, with more than 80% of newborns developing some degree of jaundice. Although most cases are physiological and self-limiting, excessive unconjugated bilirubin can cross the blood-brain barrier and result in acute bilirubin encephalopathy and, in severe cases, kernicterus. Early identification of neonates at risk and timely treatment are therefore essential to prevent bilirubin-induced neurological injury. Current guidelines for infants born at ≥35 weeks of gestation recommend that treatment decisions incorporate gestational age, postnatal age in hours, total serum bilirubin concentration, and recognized neurotoxicity risk factors [1].

Phototherapy remains the mainstay of treatment for clinically significant unconjugated neonatal hyperbilirubinemia. Blue light, particularly at wavelengths of approximately 460-490 nm, converts bilirubin into more water-soluble photoisomers that can be eliminated without hepatic conjugation [1,2]. Although phototherapy is highly effective and generally considered safe, several short-term adverse effects have been reported, including alterations in body temperature, increased insensible water loss, feeding disturbances, and biochemical abnormalities. Among these, a reduction in ionized calcium (iCa) has attracted particular attention. However, the reported frequency of phototherapy-associated hypocalcemia varies considerably. Panneerselvam K et al. reported hypocalcemia in 12.5% of term neonates receiving light-emitting diode (LED) phototherapy [3], whereas Poudel D et al. observed hypocalcemia in 21.3% of term and 34.4% of preterm neonates after 48 hours of phototherapy [4]. A more recent systematic review and meta-analysis estimated a pooled prevalence of 26.1% among term neonates, although the certainty of the available evidence was considered low [5].

The mechanism underlying phototherapy-associated reductions in iCa remains incompletely understood. Earlier hypotheses proposed that transcranial illumination suppresses pineal melatonin secretion, thereby influencing cortisol-mediated calcium homeostasis. Subsequent studies have suggested a more complex and potentially multifactorial mechanism involving alterations in parathyroid hormone, magnesium, and vitamin D metabolism [6]. Conversely, the absence of a consistent relationship between changes in melatonin and serum calcium in some studies suggests that melatonin suppression alone may not adequately explain this phenomenon [7]. Interpretation of the available evidence is further complicated by substantial heterogeneity in gestational age, duration and intensity of phototherapy, laboratory methods, and thresholds used to define hypocalcemia. Data concerning late-preterm neonates and the clinical relevance of post-phototherapy biochemical reductions in calcium also remain limited.

Given these uncertainties, further prospective evaluation in a well-defined neonatal population is warranted. Therefore, the present prospective cohort study aimed to evaluate changes in iCa before and after 24 hours of phototherapy among neonates born at ≥35 weeks of gestation with unconjugated hyperbilirubinemia. The primary objective was to quantify the change in iCa after 24 hours of phototherapy, while the secondary objective was to determine the proportion of neonates developing low iCa concentrations after 24 hours of phototherapy. We hypothesized that phototherapy would be associated with a measurable reduction in iCa concentration.

## Materials and methods

Study design and setting

A prospective cohort study was conducted in the Department of Pediatrics, BRD Medical College, Gorakhpur, Uttar Pradesh, India, from August 2021 to August 2022. Neonates with unconjugated hyperbilirubinemia who fulfilled the criteria for phototherapy were prospectively enrolled and evaluated before phototherapy and after 24 hours of phototherapy. The study was reported in accordance with the Strengthening the Reporting of Observational Studies in Epidemiology (STROBE) recommendations [8].

Study population

Neonates born at ≥35 weeks of gestation who were admitted or referred to the neonatal unit with unconjugated hyperbilirubinemia requiring phototherapy were assessed for eligibility. Only neonates who received phototherapy for at least 24 hours and for whom written informed consent was obtained from a parent or legal guardian were included.

Neonates were excluded if they had a history suggestive of birth asphyxia, neonatal sepsis, respiratory distress syndrome, major or life-threatening congenital malformations, hyperbilirubinemia requiring exchange transfusion, maternal antenatal phenobarbitone exposure, prolonged or difficult labor, or receipt of parenteral therapy. Neonates whose parents or legal guardians declined consent were also excluded.

Sample size

The sample size was estimated using the single-population proportion formula:

n = [Z²p(1 - p)] / d²

n = [(1.96)² × 0.096 × (1 - 0.096)] / (0.05)² ≈ 134

where n represents the required sample size, Z is the standard normal deviate corresponding to the desired confidence level, p is the expected proportion of phototherapy-associated hypocalcemia, and d is the absolute precision. A previous prospective study by Goyal M et al. reported an incidence of phototherapy-associated hypocalcemia of 9.6% among term and preterm neonates [9]. Using an expected proportion of 0.096, a 95% confidence level (Z = 1.96), and an absolute precision of 0.05, the minimum required sample size was approximately 134 neonates. To account for potentially incomplete observations and to improve the precision of the estimates, 145 eligible neonates were enrolled.

Phototherapy protocol

Phototherapy was initiated when the total serum bilirubin concentration reached the treatment threshold specified by the institutional neonatal hyperbilirubinemia protocol. Treatment decisions incorporated gestational age, postnatal age in hours, total serum bilirubin concentration, and the presence of relevant bilirubin neurotoxicity risk factors, in accordance with the principles of guideline-based management for neonates born at ≥35 weeks of gestation [1].

Phototherapy was administered using an LED phototherapy unit (Bird Meditech, Maharashtra, India) and was continued according to the institutional protocol. All included neonates received 24 hours of phototherapy before the post-treatment biochemical assessment.

Data collection

Demographic, perinatal, and clinical information was recorded prospectively using a structured data collection form. Variables included gestational age, sex, birth weight, postnatal age at presentation, relevant maternal and perinatal characteristics, and clinical details related to neonatal jaundice and phototherapy.

Venous blood samples were collected immediately before the initiation of phototherapy and again after 24 hours of phototherapy. Pre- and post-phototherapy biochemical measurements were performed using the same laboratory methods to minimize analytical variability.

Laboratory measurements

Blood samples were obtained from each enrolled neonate immediately before the initiation of phototherapy and again after 24 hours of phototherapy for biochemical assessment. The same analytical methods were used for the pre-phototherapy and 24-hour post-phototherapy measurements.

Total serum bilirubin was measured using the diazo method. iCa was measured using an ion-selective electrode (ISE) method. Serum sodium, potassium, and chloride were also measured using ISE methodology. All biochemical analyses were performed in the institutional laboratory according to standard operating procedures. For analysis, iCa concentrations were categorized as <1.20 mmol/L, 1.20-1.40 mmol/L, and >1.40 mmol/L.

For the prespecified study analysis, an iCa concentration <1.20 mmol/L was categorized as low. This cutoff was used as an analytical threshold rather than as a universal definition of clinically significant neonatal hypocalcemia because neonatal iCa reference intervals vary with postnatal age, gestational maturity, and analytical methodology [10].

Study outcomes

The primary outcome was the change in iCa concentration after 24 hours of phototherapy among neonates born at ≥35 weeks of gestation with unconjugated hyperbilirubinemia.

The study specifically evaluated the distribution of iCa concentrations before phototherapy and after 24 hours of phototherapy, as well as the proportion of neonates with an iCa concentration <1.20 mmol/L after 24 hours of phototherapy.

Secondary biochemical outcomes included changes in total serum bilirubin and other measured electrolytes following phototherapy.

Statistical analysis

Continuous variables were summarized as mean ± SD when approximately normally distributed and as median with IQR when non-normally distributed. Categorical variables were presented as frequencies and percentages.

Because biochemical measurements before phototherapy and after 24 hours of phototherapy were obtained from the same neonates, paired statistical methods were used. Pre- and post-phototherapy continuous measurements were compared using the paired-samples t-test when the distribution of paired differences was approximately normal.

Ionized calcium was additionally classified into three categories (<1.20, 1.20-1.40, and >1.40 mmol/L). Changes in the distribution of these paired categorical measurements before phototherapy and after 24 hours of phototherapy were assessed using the Stuart-Maxwell test of marginal homogeneity.

Statistical analyses were performed using IBM SPSS Statistics for Windows, version 23.0 (IBM Corp., Armonk, NY, USA). All statistical tests were two-sided, and a p-value <0.05 was considered statistically significant. Effect estimates were reported with 95% CIs where appropriate.

Ethical considerations

The study was initiated after approval from the Institutional Human Ethics Committee of BRD Medical College, Gorakhpur, Uttar Pradesh, India (IHEC/223(N)/2021). Written informed consent was obtained from a parent or legal guardian before enrollment. Participant confidentiality was maintained throughout data collection, analysis, and reporting.

## Results

Among 145 neonates, 64 (44.1%) were <37 weeks and 81 (55.9%) were ≥37 weeks. Mean gestational age was 36.93 ± 1.49 weeks (range 35-40 weeks). Eighty-two (56.6%) participants were male. Most neonates were delivered vaginally (109, 75.2%). Birth weight was 1.5-2.5 kg in 79 (54.5%) and >2.5 kg in 61 (42.1%). ABO incompatibility was present in 16 (11.0%) and Rh incompatibility in 2 (1.4%) neonates (Table 1).

**Table 1:** Baseline characteristics of the study participants (n = 145). kg: Kilogram.

Characteristic	Category	n (%)
Gestational age	<37 weeks	64 (44.1)
	≥37 weeks	81 (55.9)
Birth weight	<1.5 kg	5 (3.4)
	1.5-2.5 kg	79 (54.5)
	>2.5 kg	61 (42.1)
Sex	Male	82 (56.6)
	Female	63 (43.4)
ABO incompatibility	Present	16 (11.0)
Rh incompatibility	Present	2 (1.4)
Mode of delivery	Vaginal	109 (75.2)
	Caesarean section	33 (22.8)
	Assisted vaginal delivery	3 (2.1)

Phototherapy produced the expected reduction in bilirubin. The mean total serum bilirubin decreased from 14.82 ± 2.06 mg/dL before phototherapy to 9.92 ± 1.33 mg/dL after 24 hours of phototherapy, corresponding to a mean absolute reduction of 4.90 ± 2.08 mg/dL and a mean percentage reduction of 32.1%. The paired difference was statistically significant (t = 28.3, p < 0.001) (Table 2).

**Table 2:** Total serum bilirubin before phototherapy and after 24 hours of phototherapy. mg/dL: Milligrams per deciliter. Paired-samples t-test: t = 28.3, p < 0.001.

Time point	Mean ± SD (mg/dL)	Median (IQR)	Range	t-value	p-value
Pre-phototherapy	14.82 ± 2.06	14.86 (2.89)	9.78-19.67	-	-
After 24 h of phototherapy	9.92 ± 1.33	9.87 (1.67)	6.87-13.10	-	-
Absolute reduction	-4.90 ± 2.08	-5.08 (2.69)	-11.19 to -0.90	28.3	<0.001
Percent change	-32.1% ± 11.4	-32.9% (14.5)	-59% to -9%	-	-

A marked shift toward lower iCa concentrations was observed after 24 hours of phototherapy. Before treatment, 12 (8.3%) neonates had an iCa concentration <1.20 mmol/L, 24 (16.6%) had concentrations of 1.20-1.40 mmol/L, and 109 (75.2%) had concentrations >1.40 mmol/L. After 24 hours of phototherapy, the corresponding proportions were 54 (37.2%), 61 (42.1%), and 30 (20.7%), respectively. Thus, the proportion of neonates with iCa <1.20 mmol/L increased from 8.3% before phototherapy to 37.2% after 24 hours of phototherapy, representing an absolute increase of 28.9 percentage points.

Among the 133 neonates with baseline iCa ≥1.20 mmol/L, 44 developed iCa <1.20 mmol/L after 24 hours of phototherapy, corresponding to a new-onset low iCa proportion of 33.1%.

Among the 109 neonates with baseline iCa >1.40 mmol/L, 81 (74.3%) shifted to a lower calcium category after 24 hours of phototherapy, including 25 (22.9%) who developed concentrations <1.20 mmol/L. Overall, the paired distribution of iCa categories differed significantly between the pre- and post-phototherapy assessments (Stuart-Maxwell χ² = 83.808, df = 2, p < 0.001), demonstrating a significant shift toward lower iCa concentrations after 24 hours of phototherapy (Table 3).

**Table 3:** Distribution of ionized calcium before phototherapy and after 24 hours of phototherapy (n = 145) Stuart-Maxwell test: χ² = 83.808, df = 2, p < 0.001. Values are presented as n (% of the total study population). iCa: Ionized calcium; mmol/L: Millimoles per liter.

iCa after 24 h of phototherapy	Pre-phototherapy <1.20 mmol/L	Pre-phototherapy 1.20-1.40 mmol/L	Pre-phototherapy >1.40 mmol/L	Total, n (%)	Stuart-Maxwell test (χ²)	p-value
<1.20 mmol/L	10 (6.9%)	19 (13.1%)	25 (17.2%)	54 (37.2%)	83.808	<0.001
1.20-1.40 mmol/L	2 (1.4%)	3 (2.1%)	56 (38.6%)	61 (42.1%)		
>1.40 mmol/L	0 (0.0%)	2 (1.4%)	28 (19.3%)	30 (20.7%)		
Total, n (%)	12 (8.3%)	24 (16.6%)	109 (75.2%)	145 (100%)	-	-

## Discussion

The present prospective cohort study demonstrated a marked downward shift in iCa after 24 hours of phototherapy among neonates born at ≥35 weeks of gestation with unconjugated hyperbilirubinemia. The proportion of neonates with iCa <1.20 mmol/L increased from 8.3% before phototherapy to 37.2% after 24 hours of treatment, while the proportion with iCa >1.40 mmol/L decreased from 75.2% to 20.7%. The overall redistribution across paired iCa categories was highly significant (Stuart-Maxwell χ² = 83.808, p < 0.001). Importantly, among the 133 neonates who did not have low iCa at baseline, 44 developed iCa <1.20 mmol/L after 24 hours of phototherapy, corresponding to a new-onset low-iCa proportion of 33.1%. These findings indicate that phototherapy was associated with a substantial biochemical alteration in calcium homeostasis within a relatively short period of exposure.

The reduction in calcium observed in the present study is consistent with several previous investigations, although the reported frequency of hypocalcemia has varied substantially. Panneerselvam K et al. reported hypocalcemia in 12.5% of 104 term neonates receiving LED phototherapy [3]. Poudel D et al. subsequently demonstrated reductions in calcium after 48 hours of phototherapy in both term and late-preterm neonates, with hypocalcemia occurring in 21.3% and 34.4%, respectively [4]. The higher frequency among preterm neonates in that study is particularly relevant to the present cohort, in which 44.1% of participants were born before 37 completed weeks of gestation.

Studies evaluating shorter phototherapy exposure also support an early effect on calcium homeostasis. Khan M et al. studied jaundiced term neonates receiving phototherapy and reported a significant frequency of hypocalcemia, supporting the need to consider calcium alterations during treatment [11]. Vigneshwar NK et al., in an observational study of 89 neonates exposed to a median of approximately 24 hours of phototherapy, demonstrated a statistically significant reduction in mean serum calcium from 9.6 ± 0.8 mg/dL to 9.4 ± 0.6 mg/dL, although asymptomatic hypocalcemia occurred in only 2.2% [7]. The substantially greater proportion of low iCa in our cohort may reflect differences in gestational age distribution, baseline calcium status, laboratory methodology, the use of ionized rather than total calcium, and the threshold selected to define low calcium.

Other studies have reported intermediate frequencies after longer exposure. In a study of 128 full-term neonates with normal baseline serum calcium, hypocalcemia occurred in 20.3% after 48 hours of continuous conventional phototherapy; however, clinical manifestations were present in only 2.3% of the total cohort [12]. This distinction between biochemical and clinically symptomatic hypocalcemia is important when interpreting the present findings. Although 37.2% of our neonates had iCa <1.20 mmol/L after 24 hours, clinical manifestations were not systematically assessed in relation to the biochemical abnormalities; therefore, this percentage should not be interpreted as the frequency of symptomatic hypocalcemia.

The variability among published studies is further illustrated by investigations using iCa specifically. A prospective observational study of 80 term neonates reported that 58.75% experienced some reduction in iCa following phototherapy, although only 2.5% met that study's definition of hypocalcemia, and none developed symptomatic hypocalcemia [13]. Earlier work by Sethi H et al. reported considerably higher frequencies of phototherapy-associated hypocalcemia, with reductions in both total and iCa being more pronounced in phototherapy-exposed neonates than in untreated controls [14]. Taken together, these findings emphasize that the reported prevalence depends strongly on gestational maturity, phototherapy duration and intensity, the calcium fraction measured, assay methodology, and the biochemical cutoff used to define hypocalcemia.

The recent systematic review and meta-analysis by Molatefi R et al. provides broader support for an association between phototherapy and altered mineral homeostasis [5]. Across 22 studies involving 2,406 participants, phototherapy was associated with a significant reduction in serum calcium, with a pooled mean difference of -0.57 mg/dL. The pooled prevalence of post-phototherapy hypocalcemia was 26.1% (95% CI: 20.8%-32.3%). However, substantial heterogeneity was present, and the certainty of evidence for calcium outcomes was rated as low. The 37.2% prevalence of low iCa after phototherapy in our cohort is therefore above the pooled estimate, although direct numerical comparison is limited because the meta-analysis predominantly evaluated total serum calcium in term neonates, whereas the present study measured iCa and included both term and late-preterm neonates.

Gestational maturity may be an important contributor to the observed variation. Nearly half of our participants were born before 37 weeks of gestation, with a mean gestational age of 36.93 ± 1.49 weeks. Poudel D et al. found a greater absolute reduction in serum calcium among preterm than term neonates after 48 hours of phototherapy (1.16 versus 0.87 mg/dL), with hypocalcemia occurring in approximately one-third of preterm neonates [4]. Late-preterm neonates may have less mature endocrine and mineral-regulatory mechanisms and therefore may be more susceptible to disturbances in calcium homeostasis. Nevertheless, because the present analysis was not designed to establish gestational age as an independent predictor of low iCa, this explanation should be considered biologically plausible rather than demonstrated by our data.

Measurement of iCa is an important feature of the present study. Ionized calcium represents the physiologically active component of circulating calcium and, unlike total calcium, is not directly affected by serum albumin concentration. Consequently, changes in iCa may provide a more direct indication of biologically relevant calcium alterations. However, differences in neonatal iCa reference intervals and thresholds complicate comparisons across studies. In the present study, <1.20 mmol/L was used as the prespecified analytical threshold for low iCa rather than as a universal definition of clinically significant neonatal hypocalcemia [10]. This distinction is important when comparing our findings with those of studies that used total calcium or substantially different iCa thresholds.

The biological mechanism responsible for phototherapy-associated calcium reduction remains incompletely established. One traditional hypothesis proposes that transcranial phototherapy suppresses pineal melatonin secretion. Reduced melatonin activity may permit increased cortisol-mediated calcium uptake by bone, thereby lowering circulating calcium concentrations. Studies examining head covering during phototherapy have provided some support for a possible light-mediated mechanism. In one randomized study of 112 neonates, the reduction in iCa after 48 hours was greater among infants undergoing phototherapy without head covering than among those whose heads were covered, although the difference in the incidence of hypocalcemia was not statistically significant [15]. These findings are intriguing but do not establish melatonin suppression as the sole mechanism.

Indeed, more recent evidence suggests a multifactorial pathway. Beser E et al. evaluated 166 term neonates and demonstrated significant reductions in iCa, total calcium, corrected calcium, magnesium, 25-hydroxyvitamin D, and parathyroid hormone (PTH) following phototherapy [6]. Their findings support a potential contribution from suppression or alteration of parathyroid and mineral-regulatory pathways. Conversely, Vigneshwar NK et al. found only a small reduction in serum calcium after approximately 24 hours of phototherapy and no significant correlation between changes in calcium and melatonin concentrations [7]. Collectively, these observations suggest that melatonin suppression alone is unlikely to explain phototherapy-associated calcium changes and that PTH, magnesium, vitamin D, gestational maturity, and other regulatory mechanisms may also contribute.

A notable strength of the present study was the paired assessment of iCa in the same neonates immediately before phototherapy and after a standardized 24-hour exposure. This approach reduces between-subject variability and permits direct evaluation of within-neonate biochemical changes. The transition matrix also provides information that would be lost by comparing only marginal prevalences. Among 109 neonates with baseline iCa >1.40 mmol/L, 81 (74.3%) shifted to a lower category after 24 hours: 56 moved to 1.20-1.40 mmol/L and 25 to <1.20 mmol/L. Thus, the observed effect was not confined to infants crossing a single biochemical threshold but represented a broader downward redistribution of iCa.

The clinical significance of this biochemical shift requires cautious interpretation. The post-phototherapy prevalence of iCa <1.20 mmol/L was 37.2%, but 12 neonates were already below this threshold before phototherapy. Therefore, the more appropriate estimate of new-onset low iCa is 44 of 133 neonates (33.1%) who had baseline iCa ≥1.20 mmol/L. Furthermore, previous studies have shown that biochemical hypocalcemia following phototherapy is frequently asymptomatic. For example, only 2.3% of the 128 neonates studied by Shrestha S et al. had clinical signs despite biochemical hypocalcemia in 20.3% [12], while Vigneshwar NK et al. reported asymptomatic hypocalcemia in 2.2% after approximately 24 hours [7]. Therefore, our biochemical findings cannot be assumed to represent clinically important hypocalcemia in every affected neonate.

These findings should also be interpreted within contemporary neonatal practice. Current hyperbilirubinemia guidelines focus primarily on accurate bilirubin measurement, assessment of neurotoxicity risk factors, appropriate phototherapy thresholds, and monitoring of therapeutic response [1,2]. They do not establish routine calcium measurement as a requirement for every neonate undergoing phototherapy. Moreover, although the recent meta-analysis confirms a significant average reduction in calcium, the certainty of evidence was low, and the authors concluded that current evidence does not support routine supplementation for all treated neonates [5]. Accordingly, the present findings should not be interpreted as evidence for universal prophylactic calcium supplementation. Rather, they support further investigation to identify neonates at increased risk who may benefit from selective biochemical monitoring.

The present study has several strengths, including its prospective design, relatively large sample of 145 neonates, inclusion of both term and late-preterm infants, standardized assessment after 24 hours of phototherapy, paired biochemical measurements, and measurement of physiologically active iCa rather than total calcium alone. Nevertheless, several limitations should be acknowledged. First, this was a single-center observational study, which may limit generalizability. Second, the absence of a contemporaneous untreated comparison group prevents attribution of the observed decline exclusively to phototherapy. Third, potentially relevant determinants such as gestational age, birth weight, feeding status, baseline iCa, phototherapy irradiance, and other neonatal characteristics were not comprehensively evaluated as predictors of post-phototherapy low iCa. Fourth, clinical manifestations were not systematically assessed in relation to iCa concentrations. Finally, PTH, magnesium, vitamin D, and melatonin were not measured, preventing direct evaluation of the mechanisms underlying the observed biochemical changes.

Future multicenter prospective studies should use standardized definitions of neonatal hypocalcemia, measure iCa using comparable analytical methods, and precisely document phototherapy device characteristics, irradiance, and exposure duration. Stratification by gestational age may clarify whether late-preterm neonates are particularly susceptible. Concurrent assessment of iCa, magnesium, PTH, vitamin D, and clinical manifestations would help distinguish transient biochemical changes from clinically meaningful hypocalcemia. Controlled studies would also help determine whether targeted iCa monitoring in selected high-risk neonates improves clinically relevant outcomes.

## Conclusions

Phototherapy was associated with a significant downward shift in iCa among neonates born at ≥35 weeks of gestation with unconjugated hyperbilirubinemia, with the proportion of neonates having iCa <1.20 mmol/L increasing from 8.3% before treatment to 37.2% after treatment. Among neonates without low iCa at baseline, 33.1% developed concentrations <1.20 mmol/L after 24 hours of phototherapy. These findings highlight a potentially important biochemical alteration during phototherapy; however, further studies are needed to determine its clinical significance and identify neonates who may benefit from targeted calcium monitoring.
